# Editorial: Pain management in spine surgery

**DOI:** 10.3389/fsurg.2026.1862516

**Published:** 2026-05-29

**Authors:** Youbin Yang, Felicity Han, Hongfei Xiang, Ji Tu, Sidong Yang

**Affiliations:** 1 Department of Joint Surgery, The First Hospital of Hebei Medical University, Shijiazhuang, Hebei, China; 2 School of Biomedical Sciences, Faculty of Medicine, The University of Queensland, Brisbane, Queensland, Australia; 3 Department of Orthopedics, The Affiliated Hospital of Qingdao University, Qingdao, Shandong, China; 4 St George and Sutherland Clinical Campuses, University of New South Wales, Kogarah, NSW, Australia; 5 Department of Spine Surgery, Hebei Medical University Third Hospital, Shijiazhuang, Hebei, China

**Keywords:** analgesia, lumbar disc herniation, pain, spine, surgery

Spine surgery encompasses a broad spectrum of procedures, ranging from minimally invasive decompressions to complex reconstructive interventions. Despite continuous advances in surgical techniques and perioperative care, postoperative pain remains a central determinant of patient recovery, functional restoration, and overall satisfaction. Inadequately controlled pain may delay mobilization, prolong hospitalization, and increase the risk of complications, ultimately affecting both short-term outcomes and long-term quality of life.

Historically, opioid-based regimens have formed the cornerstone of postoperative analgesia in spine surgery. However, growing awareness of opioid-related adverse effects—including tolerance, dependence, and systemic complications—has prompted a shift toward multimodal and opioid-sparing strategies. Contemporary perioperative practice increasingly integrates minimally invasive surgical techniques, regional and interventional analgesia, structured rehabilitation, and individualized risk assessment to optimize recovery pathways.

Against this evolving clinical landscape, pain management in spine surgery can no longer be viewed as an isolated postoperative concern. Rather, it represents a comprehensive, perioperative framework that spans surgical planning, intraoperative optimization, early mobilization, complication prevention, and long-term functional outcomes. This Research Topic was conceived to explore and consolidate emerging evidence across these interconnected domains, with the aim of advancing safer, more effective, and patient-centered approaches to perioperative pain control in spine surgery. It provides a focused platform for examining contemporary strategies in perioperative pain management. Rather than addressing analgesia as a single intervention, it capture the multifaceted nature of perioperative pain control, encompassing surgical techniques, anesthetic and interventional approaches, risk stratification, rehabilitation strategies, and patient-reported outcomes.

The contributions collected in this issue reflect the growing recognition that effective pain management is inseparable from overall perioperative optimization. By integrating clinical investigations, predictive modeling, minimally invasive techniques, and rehabilitative interventions, this Research Topic offers a comprehensive perspective on how pain control strategies can enhance recovery, improve satisfaction, and reduce complications across diverse spine procedures.

Collectively, the studies included in this Research Topic illustrate how perioperative optimization and pain management are deeply intertwined across the continuum of spine care. Several contributions emphasize the role of minimally invasive surgical strategies in reducing tissue trauma, accelerating mobilization, and improving early postoperative pain outcomes. The study evaluating percutaneous kyphoplasty as a one-day surgical procedure demonstrated that carefully selected patients can achieve comparable safety and efficacy to traditional inpatient management, while benefiting from shorter hospitalization and improved satisfaction. Similarly, comparative analyses of full-endoscopic decompression surgery, unilateral biportal endoscopic discectomy in geriatric patients, and matched cohort comparisons between percutaneous endoscopic lumbar discectomy and microendoscopic discectomy highlight the growing importance of surgical approaches that minimize soft tissue disruption and facilitate faster functional recovery. Technical refinements, such as radiographic criteria guiding rib resection during minimally invasive thoracolumbar corpectomy, further reflect a broader commitment to reducing operative morbidity and postoperative discomfort.

Beyond surgical technique, several studies address direct analgesic and interventional strategies aimed at optimizing perioperative pain control. The randomized double-blind controlled trial assessing transcutaneous electrical nerve stimulation and trans-auricular vagus nerve stimulation during monitored anesthesia care underscores the expanding interest in non-pharmacological neuromodulation techniques as adjuncts to anesthesia. Interventional approaches, including modified transforaminal epidural steroid injection combined with pulsed radiofrequency, demonstrated sustained pain relief and functional improvement in patients with lumbar radiculopathy. Meanwhile, perioperative pharmacologic optimization is reflected in the comparison of epsilon-aminocaproic acid and tranexamic acid during posterior lumbar interbody fusion, illustrating how blood management strategies may indirectly influence recovery trajectories and postoperative comfort.

Risk stratification and outcome prediction represent another important theme within this Topic. The development and validation of a nomogram-based model to predict residual back pain following percutaneous kyphoplasty highlights the value of individualized perioperative assessment. Similarly, analyses identifying risk factors for postoperative dissatisfaction after transforaminal lumbar interbody fusion, as demonstrated in a single-center study evaluating predictors of dissatisfaction after TLIF, together with large-scale evaluations of recurrence risks and clinical outcomes examining the safety and effectiveness of TLIF surgery for lumbar disc herniation, reinforce the concept that postoperative pain and satisfaction are multifactorial phenomena influenced by biological, psychological, and procedural variables.

Complementing surgical and predictive approaches, rehabilitation and complication management play critical roles in postoperative recovery. The study investigating rehabilitation gymnastics combined with compression therapy in elderly patients significantly improved early functional outcomes and reduced complication rates, underscoring the value of coordinated postoperative programs. Analyses of postoperative lumbar intervertebral space infections following spinal endoscopy and rare but severe complications such as dural tears with cauda equina herniation after percutaneous endoscopic lumbar discectomy emphasize the importance of vigilant monitoring and timely intervention. In addition, imaging-based investigations into the potential protective effects of aspirin on lumbar degeneration provides further insight into long-term structural and pain-related considerations.

Taken together, the contributions in this Research Topic demonstrate that effective pain management in spine surgery extends far beyond pharmacological analgesia alone. It encompasses minimally invasive technique selection, perioperative blood management, interventional neuromodulation, individualized risk assessment, structured rehabilitation, and proactive complication prevention. This integrated perspective reflects a shift toward comprehensive perioperative frameworks that aim not only to reduce pain intensity, but also to enhance recovery pathways, improve patient satisfaction, and optimize long-term functional outcomes. Looking forward, the evolution of pain management in spine surgery will likely depend on deeper integration of individualized risk assessment, minimally invasive techniques, and multidisciplinary perioperative pathways. Predictive modeling tools may increasingly guide patient selection and preoperative counseling, enabling more personalized recovery strategies. At the same time, continued refinement of endoscopic and motion-preserving procedures holds promise for reducing surgical trauma and accelerating functional restoration. Non-pharmacological neuromodulation, targeted interventional therapies, and structured rehabilitation programs may further complement pharmacologic approaches in achieving sustainable pain control while minimizing reliance on opioids.

Future investigations should also focus on long-term functional outcomes, patient-reported measures, and the interaction between psychological, biological, and procedural factors influencing recovery. As healthcare systems increasingly emphasize value-based care, optimizing pain management strategies will require not only effective analgesia, but also cost-efficiency, safety, and durable patient satisfaction. Through continued collaboration across surgical, anesthetic, rehabilitative, and research disciplines, pain management in spine surgery can progressively shift toward more precise, patient-centered, and outcome-driven frameworks.

